# Supplementary material for: Labile Photo‐Induced Free Radical in α‐Ketoglutaric Acid: a Universal Endogenous Polarizing Agent for In Vivo Hyperpolarized 13C Magnetic Resonance
Source: Angew Chem Weinheim Bergstr Ger. 2021 Nov 25;134(2):e202112982. doi: 10.1002/ange.202112982 (PMC10947361; doi:10.1002/ange.202112982)
Supplement: Supplementary file 1 — Supporting Information [file ANGE-134-0-s001.pdf]

Angewandte Chemie
Eine Zeitschrift der Gesellschaft Deutscher Chemiker

Supporting Information

Labile Photo-Induced Free Radical in α-Ketoglutaric Acid: a Universal Endogenous Polarizing Agent for In Vivo Hyperpolarized ^{13}C Magnetic Resonance

*Adam P. Gaunt, Jennifer S. Lewis, Friederike Hesse, Tian Cheng, Irene Marco-Rius, Kevin M. Brindle, and Arnaud Comment**

ange_202112982_sm_miscellaneous_information.pdf

Supporting Information

Experimental section

Sample preparation

Samples were prepared by dissolving either 75 mM or 300 mM of solid α-KG in a 80:20 (w:w) [1-^{13}C]lactic acid:water (w:w) solution (Sigma Aldrich, Gillingham, UK) to give a 8.88 M lactic acid concentration. Vitrified beads were produced by flash freezing droplets (6 μL) of solution in liquid nitrogen. The beads were placed in the 5-mm outer diameter tail of a quartz Dewar (Wilmad 150 mL Suprasil Dewar Flask type WG-850-Q) prefilled with liquid nitrogen and irradiated with UV-Vis light using a Dymax Bluewave200 broadband source (Dymax, Wiesbaden, Germany). A 200 W bulb provides a broadband UV source from 280 to 450 nm and the sample receives 40 W/cm2 of light, delivered through a 5 mm diameter circular light guide. The beads were irradiated 4 at a time and the resulting radical-containing beads stored in liquid nitrogen.

X-band ESR measurements at 77K

ESR spectra of the frozen beads were measured at 77 K with a 9.4 GHz benchtop ESR spectrometer (Miniscope 400, Magnettech, Berlin, Germany) using the same 5-mm outer diameter tail quartz Dewar used for UV-Vis irradiation, The radical concentration was determined by comparing the double integral of the ESR spectrum to a predefined calibration curve, created from a set of known concentrations of the persistent radical compound, 4-hydroxy-2,2,6,6-tetramethylpiperidine-N-oxyl (TEMPOL; molar mass 172.24 g/mol) dissolved in H_2O (w:w).

Liquid chromatography high resolution mass spectrometry of melted photo-irradiated sample

A sample prepared from a photo-irradiated frozen 300 mM α-KG dissolved in water was analyzed using a Shimadzu Nexera X2 UHPLC coupled to a Sciex TripleTOF® 6600 mass spectrometer. Chromatographic separation was achieved using a SeQuant ZIC-pHILIC 5µm 150 x 2.1mm column (and Sequant ZIC-pHILIC 20 x 2.1mm guard column) at 45°C. The mass spectrometer was operated in negative ion mode. Data were acquired using Sciex Analyst® TF software. Gradient elution was performed with 100% Acetonitrile and an increasing proportion of 20 mM Ammonium Carbonate pH 9.4, over 17 minutes at 200 µL/min. This enabled the retention and separation of the metabolites of interest, with a further

11.5 minutes of column re-equilibration. Extracted ion chromatograms were processed using the theoretical accurate mass ±20 ppm and integrated using Sciex MultiQuant software. The results are displayed in Fig. S1.

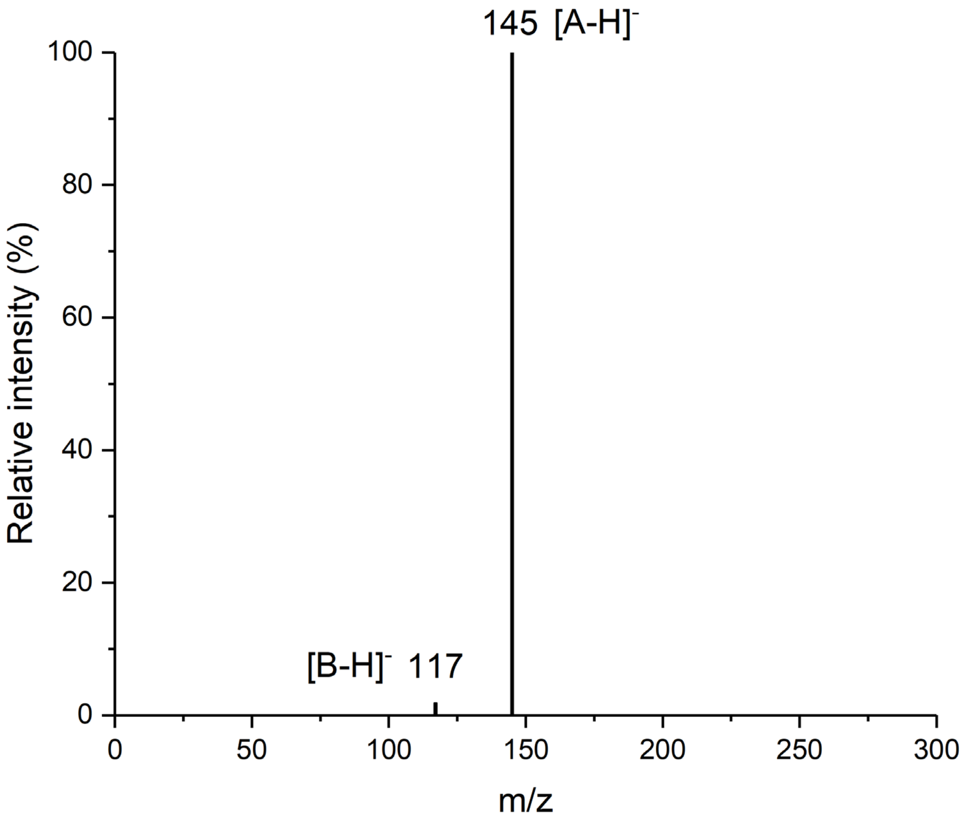

Figure S1.. Liquid chromatography high resolution mass spectrometry showing the presence of the precursor α-KG (A) and the byproduct succinic acid (B).

^{13}C liquid-state MR analysis of melted photo-irradiated samples

^{13}C MR measurements were made using a 600 MHz Avance spectrometer (Bruker, Billerica, MA, USA). Frozen 6 µL beads (vitrified droplets of sample) containing 300 mM [1-^{13}C]α-KG in 80:20 (w:w) [1-^{13}C]lactic acid:water (w:w) were photo-irradiated for 30 s, or non-irradiated (controls), and then dissolved in 400 µL of water, containing 2 mM gadopentetic acid dimeglumine salt (Magnevist, Bayer). ^{13}C MR spectra were acquired using a 90° pulse, a repetition time of 7 s and a spectral width of 108 ppm and were the sum of 1024 transients.

Sample loading and DNP

DNP was carried out as described previously using a zero boil-off DNP polarizer operating at 7 T and 1.35K[1] with a 20 mW 197 GHz ELVA microwave source, with a built-in voltage controlled frequency oscillator. The performance of this polarizer is similar to the system developed by Jähnig *et al.*[2] A custom-designed fluid path assembly similar, to the one described previously, was used to hyperpolarize the samples but the sample vial was adapted for loading the beads containing labile photo-irradiated free radicals. The bottom part of the vial was machined in polyether ether ketone (PEEK) with a long thread (9 mm) which, once the sample had been introduced into the vial, could be screwed to a PEEK vial top in a leak-tight manner using a single-use polytetrafluoroethylene (PTFE) O-ring seal (Fig.S2). To load samples without quenching the labile photo-induced radicals, the bottom half of the two-part sample vial was precooled and then partially submerged in liquid nitrogen until the vial was closed. Helium gas was flushed at low pressure (1 bar starting pressure) through the fluid path throughout the loading process to prevent the introduction of air into the lumen. When sealed the vial was rapidly loaded into the polarizer using a procedure that maintains the temperature of the sample below 100 K (see Fig.S3).

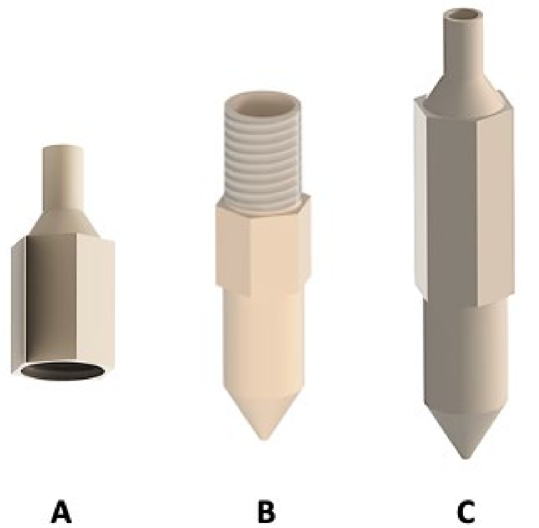

Figure S2. Two-part screw sample vial. (a) The vial top, which is glued to the outer lumen of the fluid path with epoxy resin, (b) the vial bottom in which the sample is loaded, and (c) the fully assembled vial ready for insertion in the hyperpolarizer. The vial bottom is designed to be held by the hex section in a container filled with liquid nitrogen to maintain the sample below 100 K during the loading process.

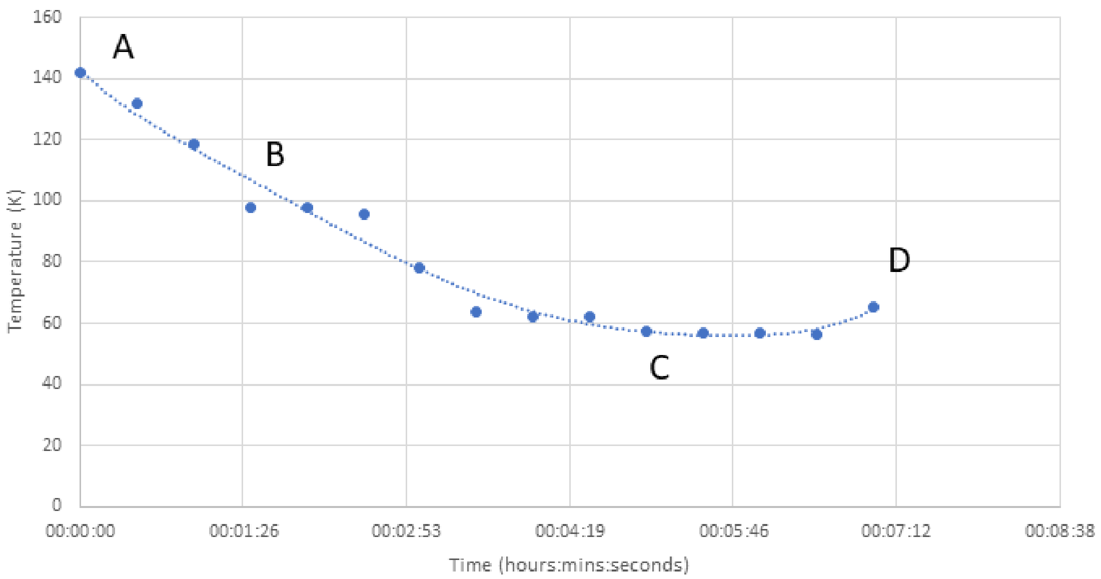

Figure S3. Temperature profile measured during sample loading using a Pt100 temperature sensor affixed inside the vial top. (A) Once the sample has been introduced inside the vial bottom pre-cooled in a liquid nitrogen bath, the vial top is cooled using the cold nitrogen gas evaporating from the liquid nitrogen bath. A flow of helium gas (1 bar) is directed through the inner lumen of the fluid path during the procedure. (B) The vial bottom is screwed onto the vial top while maintaining the helium gas flow until (C) the fluid path is closed, trapping helium gas inside the fluid path. (D) The vial is then transferred to the airlock of the polarizer and quickly lowered inside the sample space of the polarizer, at the level of the 50-K plate[1]. ESR experiments carried out before and after loading confirmed that the radical concentration had not been affected by the process of loading and unloading the sample.

Solid-state DNP experiments were performed in a photo-irradiated 80:20 [1-^{13}C]lactic acid:water (w:w) sample containing 300 mM [1-^{13}C]α-KG to find the optimum microwave frequency with and without microwave frequency modulation. The sweep was carried out from 196.7 to 197.2 GHz. At each microwave frequency, the maximum solid-state ^{13}C MR signal was measured after a polarization time of 60 min using a 90-degree RF pulse. A 4 s ^{13}C radiofrequency saturation pulse was applied prior to setting a new microwave frequency. The optimal modulation parameters were 1.2 KHz for the frequency and 52 MHz (4 V) for the amplitude. These results confirm what has already been observed by Hovav *et al.* for wide ESR line free radicals[3]: less and less electron spins can be excited by monochromatic microwave irradiation and modulating the frequency allows exciting a larger portion of the ESR line, which greatly enhances DNP. Electron spin relaxation time is also a crucial parameter for the efficiency of the microwave irradiation, but correlating it to the optimal microwave frequency modulation parameter is beyond the scope of this work.

The maximum solid-state ^{13}C polarization estimated by comparing the ^{13}C signal after 3 h of microwave irradiation with the signal from a concentrated standard (1 mL of 90% [1-^{13}C]lactic acid aqueous solution) at thermal equilibrium was 40 ± 10 %.

Liquid state HP ^{13}C MR measurements

Four 6 µL frozen beads containing 300 mM [1-^{13}C]α-KG in 80:20 (w:w) [1-^{13}C]lactic acid:water (w:w) that had been photo-irradiated for 30 s were loaded in the two-part vial together with four 6 µL frozen beads of an NaOH solution. The concentration of the NaOH solution was determined prior to the dissolution experiments so as to neutralize the lactic acid to give a HP [1-^{13}C]lactate solution with a pH between 6.5 and 8.5 following dissolution. The pH was confirmed to be 7.5 + 1.0 using pH universal indicator paper. Dissolution was carried out using 5 mL of superheated D_2O (145 °C at 11 bar). The resulting HP [1-^{13}C]lactate solution was collected in 50 mL Falcon tube prior to manual injection via a PTFE capillary into a 5 mm diameter NMR tube in the bore of a vertical 600 MHz high-resolution MR system (Avance, Bruker, Germany) within 30 s of initiating the dissolution process. The HP ^{13}C signal was measured using a train of 64, 10° pulses applied every 3.5 s. The spectral bandwidth was set to 100 ppm and the center frequency set to 165 ppm. The liquid-state ^{13}C polarization was calculated by comparing the integral of the HP [1-^{13}C]lactate peak to the integral of the thermally polarized [1-^{13}C]lactate peak acquired by summing 1024 transients acquired every 15 s using the identical scan parameters to the hyperpolarized acquisition, with the addition of an increased delay between transients in order to ensure full recovery of the spin system. ^{13}C signals from the [1-^{13}C]lactate (183.2 ppm), its three impurities at 177, 177.7 and 179.2 ppm, as well as [1-^{13}C] α-KG (170.8 ppm) and [5-^{13}C] α-KG (182.3 ppm) were detected but no $^{13}CO_2$ was left in the solution due to degassing during dissolution (Fig. S4).

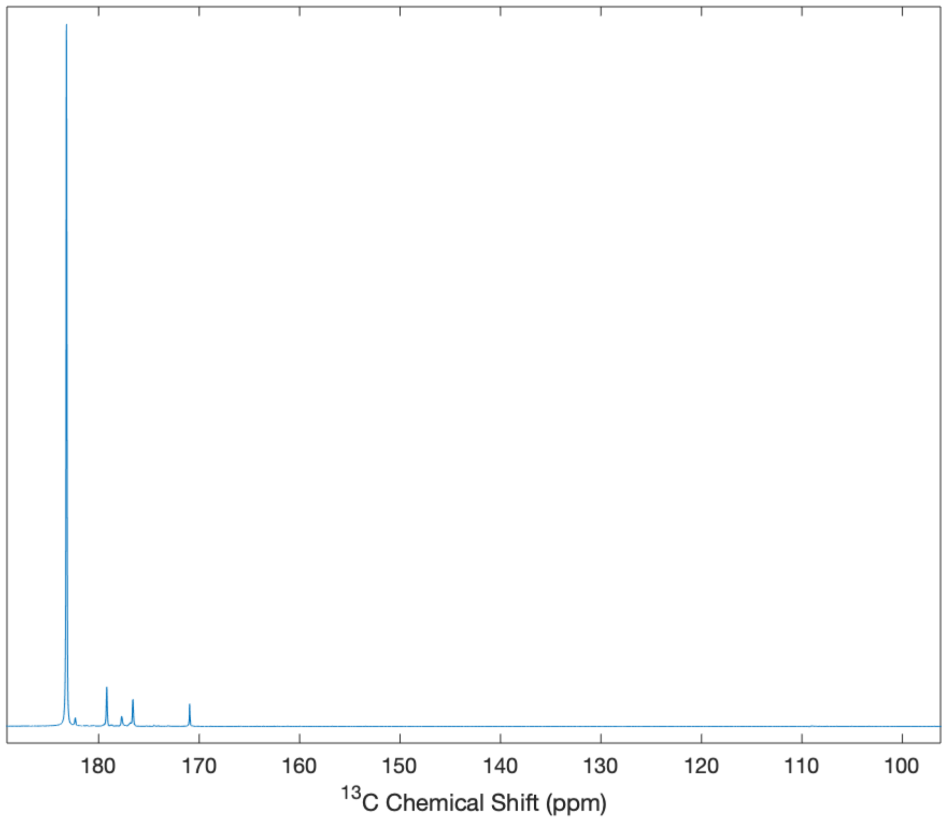

Figure S4. HP ^{13}C spectrum recorded at 14.1 T 30 s after dissioluition in 5 mL of superheated D_2O (145 °C at 11 bar) of four 6 μL frozen beads containing 300 mM α-KG in 80:20 (w:w) [1-^{13}C]lactic acid:water (w:w) that had been photo-irradiated for 30 s (radical concentration measured by ESR was 50 mM) and an equivalent amount of frozen NaOH solution. Final pH was 6.5.

Analogous experiments with α-KG instead of [1-^{13}C] α-KG were also performed to test the purity of the compounds prior to in vivo experiments (see Fig. S5). The same small 3 peaks from [1-^{13}C]lactate impurities were visible in the spectrum. The presence of these impurities in HP [1-^{13}C]lactic acid was previously reported by Lau *et al.*[4]

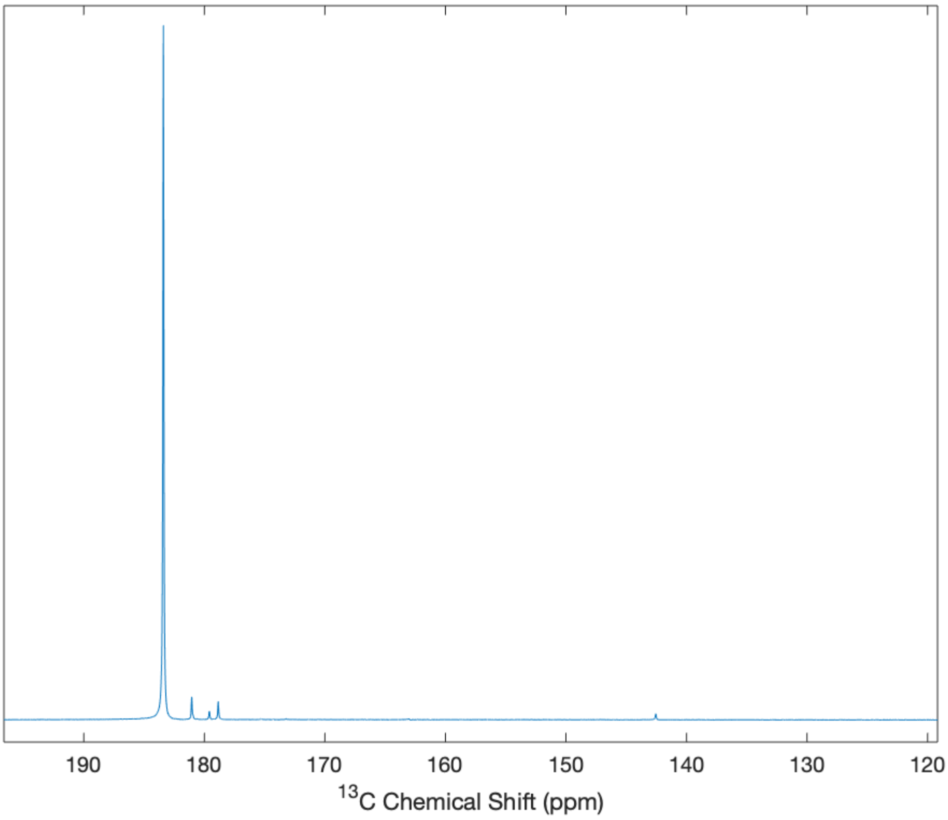

Figure S5. HP ^{13}C spectrum recorded at 14.1 T 30 s after dissolution in 5 mL of superheated D_2O (145 °C at 11 bar) of four 6 µL frozen beads containing 300 mM α-KG in 80:20 (w:w) [1-^{13}C]lactic acid:water (w:w) that had been photo-irradiated for 30 s (radical concentration measured by ESR was 50 mM) and an equivalent amount of frozen NaOH solution. Final pH was 6.5.

Animal preparation

All procedures were performed in compliance with project and personal licenses issued under the United Kingdom Animals (Scientific Procedures) Act, 1986 and were approved by the Cancer Research UK, Cambridge Institute Animal Welfare and Ethical Review Body. Female Wistar rats (350 $\pm$ 10 g) were initially anesthetized with 4% isoflurane in air/oxygen mix (3:1 v:v) A catheter was inserted into the tail vein for substrate delivery. A 30G needle connected to a 1 mL syringe containing Vetivex® and heparin (prepared previously by adding 5000 units of heparin to 100 mL of Vetivex®) was used to prevent clotting**.** The respiration rate, and temperature were monitored using a pneumatic respiration sensor, and a rectal temperature probe (Small Animal Instruments Inc, Stony Brook, NY), respectively. After insertion of the cannula the isoflurane concentration was decreased to 2.5% in air/oxygen mixture and the animals placed in a supine position in a custom-designed animal holder. Animal body temperature was maintained by a flow of warm air.

In vivo MR measurements at 9.4T

MR experiments were carried out in a 9.4 T horizontal bore magnet (Magnex Scientific, Oxford, UK) interfaced to a Bruker console (Bruker, Germany). Gradient echo ^{1}H imaging was used to ensure the correct placement of a 10 mm (outer diameter) ^{13}C transmit/receive surface coil placed over the liver of the rat. A 1 mL bolus containing 42 $\pm$ 3 mM of [1-^{13}C]lactate was injected 30 $\pm$ 3 s after the dissolution process was initiated. The bolus was injected over 10 ± 1 s, The ^{13}C MR acquisition was started simultaneously with the dissolution and consisted of 128 transients recorded using 20-degree flip angle radiofrequency pulses separated by a repetition time of 2 s. The spectral width was set to 108.16 ppm centered at 140 ppm. Residual hyperpolarized sample was collected post dissolution to measure the pH and concentration of the injected sample. The concentration was measured using ^{13}C MR by comparing the integral of the peaks to a standard sample of known concentration. Note that the dose could be possibly further reduced by using deuterated ^{13}C-lactate since it would provide larger signals due to its extended T_1.[5]

Reference

[1] T. Cheng, A. P. Gaunt, I. Marco-Rius, M. Gehrung, A. P. Chen, J. J. van der Klink, A. Comment, *NMR Biomed* **2020**, *33*, e4264.
[2] F. Jähnig, G. Kwiatkowski, A. Däpp, A. Hunkeler, B. H. Meier, S. Kozerke, M. Ernst, *Phys Chem Chem Phys* **2017**, *19*, 19196-19204.
[3] Y. Hovav, A. Feintuch, S. Vega, D. Goldfarb, *J Magn Reson* **2014**, *238*, 94-105.
[4] aT. Abe, S. Takahashi, N. Suzuki, *J Cerebr Blood F Met* **2006**, *26*, 153-160; bA. Z. Lau, A. P. Chen, C. H. Cunningham, *NMR Biomed* **2021**, *34*, e4532.
[5] C. Taglang, D. E. Korenchan, C. von Morze, J. Yu, C. Najac, S. Wang, J. E. Blecha, S. Subramaniam, R. Bok, H. F. VanBrocklin, D. B. Vigneron, S. M. Ronen, R. Sriram, J. Kurhanewicz, D. M. Wilson, R. R. Flavell, *Chem Comm* **2018**, *54*, 5233-5236.
